# Heterologous Expression of the *Constitutive Disease Resistance 2* and *8* Genes from *Poncirus trifoliata* Restored the Hypersensitive Response and Resistance of *Arabidopsis cdr1* Mutant to Bacterial Pathogen *Pseudomonas syringae*

**DOI:** 10.3390/plants9070821

**Published:** 2020-06-30

**Authors:** Xiaobao Ying, Bryce Redfern, Frederick G. Gmitter, Zhanao Deng

**Affiliations:** 1 Gulf Coast Research and Education Center, IFAS, University of Florida, 14625 County Road 672, Wimauma, FL 33598, USA; yingxb75@ufl.edu (X.Y.); killerblr@ufl.edu (B.R.); 2 Citrus Research and Education Center, IFAS, University of Florida, 700 Experiment Station Road, Lake Alfred, FL 33850, USA; fgmitter@ufl.edu

**Keywords:** Huanglongbing, disease resistance, cisgenics, genetic engineering, *Poncirus*, *Arabidopsis*

## Abstract

Huanglongbing (HLB), also known as citrus greening, is the most destructive disease of citrus worldwide. In the United States, this disease is associated with a phloem-restricted bacterium, *Candidatus* Liberibacter asiaticus. Commercial citrus cultivars are susceptible to HLB, but *Poncirus trifoliata*, a close relative of *Citrus*, is highly tolerant of HLB. Isolating *P. trifoliata* gene(s) controlling its HLB tolerance followed by expressing the gene(s) in citrus is considered a potential cisgenic approach to engineering citrus for tolerance to HLB. Previous gene expression studies indicated that the *constitutive disease resistance* (*CDR*) genes in *P. trifoliata* (*PtCDRs*) may play a vital role in its HLB tolerance. This study was designed to use *Arabidopsis* mutants as a model system to confirm the function of *PtCDRs* in plant disease resistance. *PtCDR2* and *PtCDR8* were amplified from *P. trifoliata* cDNA and transferred into the *Arabidopsis cdr1* mutant, whose resident *CDR1* gene was disrupted by T-DNA insertion. The *PtCDR2* and *PtCDR8* transgenic *Arabidopsis cdr1* mutant restored its hypersensitive response to the bacterial pathogen *Pseudomonas syringae* pv. *tomato* strain DC3000 (*Pst* DC3000) expressing *avrRpt2*. The defense marker gene *PATHOGENESIS RELATED 1* (*PR1*) expressed at much higher levels in the *PtCDR2* or *PtCDR8* transgenic *cdr1* mutant than in the non-transgenic *cdr1* mutant with or without pathogen infection. Multiplication of *Pst* DC3000 bacteria in *Arabidopsis* was inhibited by the expression of *PtCDR2* and *PtCDR8*. Our results showed that *PtCDR2* and *PtCDR8* were functional in *Arabidopsis* and played a positive role in disease resistance and demonstrated that *Arabidopsis* mutants can be a useful alternate system for screening *Poncirus* genes before making the time-consuming effort to transfer them into citrus, a perennial woody plant that is highly recalcitrant for *Agrobacterium* or biolistic-mediated transformation.

## 1. Introduction

Huanglongbing (HLB), also known as citrus greening, is the most destructive disease in citrus worldwide. The typical symptoms of HLB include asymmetrical blotchy yellowing or mottling on leaves and yellowing of leaf veins. As the disease progresses, citrus fruit become lopsided and smaller, and diseased mature fruit remain partially green. Eventually, the infected trees die [1]. HLB is associated with the phloem-restricted gram-negative bacteria *Candidatus* Liberibacter spp. that can be transmitted by insect vectors or grafting [2]. In the United States (U.S.), the presumptive pathogen of HLB is *Candidatus* Liberibacter asiaticus (*C*las), and the insect vector of *C*Las is Asian citrus psyllid (ACP) (*Diaphorina citri*). The outbreak of HLB in Florida has resulted in a 72.2% reduction in citrus juice production and a 20.5% reduction in fresh market fruit in the U.S. from 2007–2008 to 2017–2018 [3]. This disease has been responsible for substantial increases in the costs of citrus grove maintenance and management [4]. The great majority of commercial citrus cultivars are highly susceptible to HLB [5]. The pathogen has not been cultured, and the virulence mechanisms of *C*Las remain largely unknown. Nevertheless, several reports have shown that *C*Las encodes effectors to inhibit citrus plant defense and development [6,7,8,9,10,11,12,13]. 

Management of HLB has been a lofty challenge, although a wide range of management techniques have been tested, ranging from the frequent application of insecticides in an attempt to suppress the psyllid vector, enclosing individual citrus trees in ACP-proof nets to prevent them from contact with infectious psyllids [14] to planting new citrus trees inside ACP-proof screen houses and the application of antibiotics to citrus trees. So far, the primary techniques used by Florida growers for managing HLB under field conditions have been applying insecticides to suppress the population of ACP [15] and modifying nutrient management strategies to reduce HLB symptoms and improve fruit yield and quality [16,17,18,19]. These management practices have been extremely costly, making them an unsustainable endeavor. With the citrus production in Florida alone being a $9 billion industry, it is vital to find more effective, economic, and sustainable management strategies to curb the devastation caused by this disease [20]. 

The development and use of HLB-resistant/tolerant cultivars is considered the best long-term management strategy for this bacterial disease. Toward finding HLB resistance/tolerance, numerous citrus cultivars, close relatives, and distant citrus relatives have been screened under natural disease pressure and ACP presence or by artificial *C*Las inoculation. Within the genus *Citrus*, a number of commercial cultivars with HLB tolerance were identified, including ‘LB-9′ Sugar Belle^®^ mandarin, ‘Temple’ tangor, rough lemon, etc. [21,22,23,24,25]. A number of rootstock cultivars resulting from crosses between *Citrus* and *Poncirus* showed strong tolerance to HLB [26,27,28]. Several distant citrus relatives were found having strong resistance to HLB [14,29]. In these evaluations, sweet orange, grapefruit, and most mandarins, citrus of the most important commercial value to the industry, were highly sensitive to HLB. Sweet orange, grapefruit, and mandarin cultivars with strong tolerance, ideally resistance, to HLB are much needed.

To increase citrus tolerance or resistance to HLB, a number of foreign or synthetic genes have been introduced into citrus and have shown some promising results. For example, thionin, belonging to the pathogenesis-related 13 family [30], overexpressed in citrus resulted in increased resistance to HLB and citrus canker [31]. Another antimicrobial peptide, cecropin B, was expressed in citrus phloem and reduced HLB severity in the transgenic citrus [32]. The master regulator gene of plant defense, *NPR1* from *Arabidopsis*, was transferred into citrus, and the transgenic plants showed enhanced resistance to HLB [33]. Although these transgenic approaches can be a powerful tool to develop HLB resistance/tolerance, commercialization and export of transgenic citrus fruit and processed citrus products may encounter marketplace resistance because of negative public perception and anti-GMO sentiments. 

To make engineered HLB resistant/tolerant citrus cultivars more acceptable to citrus consumers and producers, cisgenic approaches seem worthy of exploration. Cisgenic approaches have been pursued in a number of crops, and cisgenic apple lines with resistance to several diseases have been developed [34,35]. Consumer surveys indicated that cisgenic products were evidently more acceptable to consumers than transgenic products [36]. Recently, the U.S. Department of Agriculture (USDA) Animal and Plant Health Inspection Service (APHIS) issued new rules and regulations governing the movement, including environmental release, of certain genetically engineered (GE) organisms (https://www.aphis.usda.gov/brs/fedregister/BRS_2020518.pdf). Under these new rules, GE plants can be exempted from regulation if the genetic modification in the plants introduces a gene known to occur in the plant’s gene pool. As these new rules and regulations become effective in 2020, citrus cultivars with genetically engineered HLB resistance from cisgenic approaches may become commercialized more readily than before in the U.S. It should be pointed out that at this time, regulatory bodies in other countries have not differentiated cisgenic from transgenic plants.

Numerous efforts have been made to understand the tolerance mechanisms of citrus with regards to HLB. Basal resistance was considered to play an important role in the tolerance of some citrus to HLB, as shown by analyzing the transcriptional profiles of two closely related HLB-tolerant ‘Jackson’ grapefruit-like hybrid trees and HLB-susceptible ‘Marsh’ grapefruit trees [37]. HLB-tolerant rough lemon showed a stronger and faster response to *C*Las infection at earlier stages than susceptible sweet orange [22]. The *constitutive disease resistance 1* (*CDR1*) has been implicated in HLB tolerance in some studies [28]. This gene was induced in HLB-susceptible ‘Cleopatra’ mandarin, but the basal expression level of *CDR1* was much higher in the HLB-tolerant citrus rootstock cultivar ‘US-897′ than in ‘Cleopatra’ mandarin [28]. Constitutive overexpression of *CDR1* in *Arabidopsis* resulted in activation of its defense genes and enhanced plant disease resistance [38]. *Arabidopsis* CDR1 is an extracellular aspartic protease with the conserved catalytic sequence motifs DTG and DSG [39]. The optimized condition for CDR1 activity is a pH of 6.0–6.5 in a dimerized state [40]. Salicylic acid (SA) was required in CDR1-mediated disease resistance [28]. Expression of the *CDR1* gene in rice (*OsCDR1*) was activated upon treatment with SA [41]. Overexpression of *OsCDR1* in *Arabidopsis* and rice conferred enhanced resistance against bacterial and fungal pathogens [41]. The function of OsCDR1 in disease resistance was found to be dependent on its proteinase activity [41,42].

*Poncirus trifoliata* is a close relative of *Citrus* [43] and has been the most important source of disease resistance genes for citrus breeding and genetic improvement. Several studies have shown that *P. trifoliata* and its hybrids with *Citrus* are highly tolerant to HLB [14,44]. It was found that the *constitutive disease resistance 2* and *constitutive disease resistance 8* from *P. trifoliata* (*PtCDR2/PtCDR8*) were upregulated upon *C*Las infection [45]. If these genes could confer citrus resistance/tolerance to HLB, they could be used in a cisgenic approach to produce HLB-resistant/tolerant citrus cultivars that may be more acceptable to citrus consumers and producers. In this study, *PtCDR2*/*PtCDR8* were cloned and transferred into the *Arabidopsis cdr1* mutant. *PtCDR2*/*PtCDR8* transgenic *Arabidopsis cdr1* lines showed a typical hypersensitive response (HR) to *Pseudomonas syringae* pv. *tomato* strain DC3000 (*Pst* DC3000) expressing *avrRpt2* (*Pst* DC3000 *avrRpt2*), a model pathogen widely used to test *Arabidopsis* plants for disease resistance. Expression of *PtCDR2* and *PtCDR8* in *Arabidopsis cdr1* lines inhibited growth of *Pst* DC3000 *avrRpt2.* Our results showed that *Arabidopsis* mutants could be used to screen genes from *Poncirus* (and *Citrus*) for their roles in plant disease resistance before extensive studies in *Citrus*. Using *Arabidopsis* mutants as an alternate system may help identify candidate genes for engineering citrus for HLB tolerance/resistance.

## 2. Results

### 2.1. Cloning and Structure of *Constitutive* Disease Resistance 2 and 8 from Poncirus trifoliata

**Figure 1:**
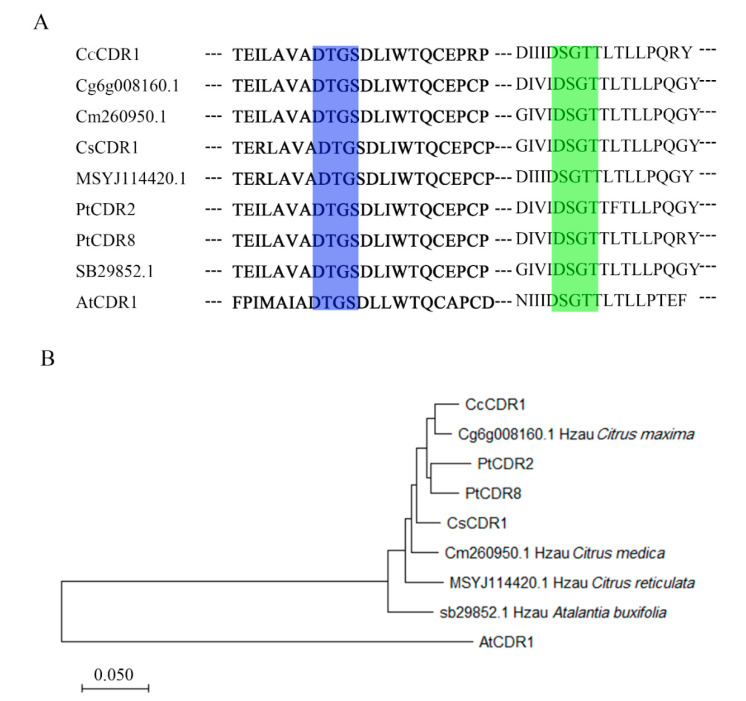
Bioinformatical analysis of PtCDR2 and PtCDR8 with CDR1s from other citrus and *Arabidopsis*. (**A**) Two conserved catalytic motifs were present in PtCDR2, PtCDR8, and CDR1s from *Arabidopsis* (AtCDR1), *Citrus maxima* (Cg6g008160.1), *Citrus medica* (Cm260950.1), *Citrus reticulata* (MSYJ114420.1), *Atalantia buxifolia* (sb29852.1), *Citrus sinensis* (CsCDR1), and *Citrus clementina* (CcCDR1). The sequences of the two conserved catalytic motifs were DTGS (in the blue box) and DSGT (in the light green box). The sequences were aligned with ClustalW. (**B**) Phylogenetic tree was generated by the neighbor-joining method using MEGA X.

*PtCDR2* and *PtCDR8* were amplified from cDNA synthesized from mRNAs isolated from mature leaves of *P. trifoliata*. The deduced PtCDR2 and PtCDR8 proteins both contain 428 amino acid residues and share 94.2% identity. PtCDR2 and PtCDR8 were aligned with predicted CDR1 proteins from sweet orange (*Citrus sinensis*) (CsCDR1 XP_006484735), Clementine mandarin (*Citrus clementina*) (CcCDR1 XP_006437356), pummelo (*Citrus maxima*) (Cg6g008160.1), citron (*Citrus medica*) (Cm260950.1), ‘Mangshan’ mandarin (*Citrus reticulata*) (MSYJ114420.1), Chinese box orange (*Atalantia buxifolia*) (sb29852.1), and *Arabidopsis* (AtCDR1 AY243479). Two conserved domains (DTGS and DSGT) were found in these deduced proteins (Figure 1A). PtCDR2 and PtCDR8 fell into the same clade in the phylogenetic tree (Figure 1B). 

### 2.2. PtCDR2 and PtCDR8 Restored the Hypersensitive Response of Arabidopsis cdr1 Mutant to Pathogen

**Figure 2:**
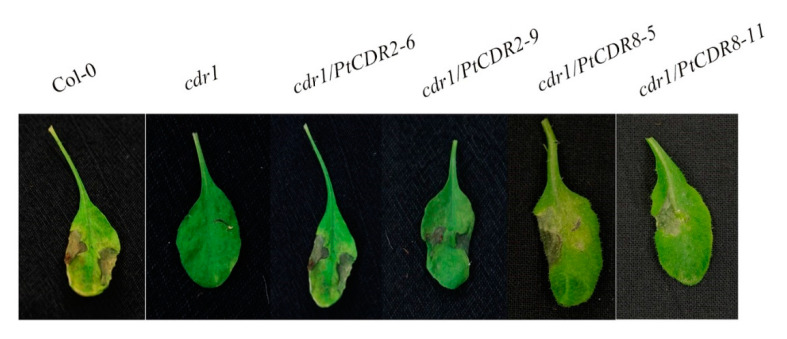
*PtCDR2* and *PtCDR8* restored hypersensitive response. Wild-type *Arabidopsis* Col-0, *cdr1* mutant, *PtCDR2* transgenic *cdr1* line 6/line *9*, and *PtCDR8* transgenic *cdr1* line 5/line 11 were infiltrated with *Pseudomonas syringae* pv. *tomato* strain DC3000 (*Pst* DC3000) expressing *avrRpt2*. Cell death or hypersensitive response (HR) was not observed in the infiltrated area of leaves of the *cdr1* mutant; wild-type *Arabidopsis*, *PtCDR2* transgenic *cdr1* lines, and *PtCDR8* transgenic *cdr1* lines all showed cell death or HR in the infiltrated leaf area.

To test whether *PtCDR2* and *PtCDR8* can function as *CDR1* in disease resistance, an *Arabidopsis cdr1* mutant resulting from T-DNA insertion [46] was transformed with *PtCDR2* and *PtCDR8* separately. Multiple T0 transgenic lines were obtained for each gene. These T0 lines were selfed, and multiple T1 transgenic lines were produced. T1 transgenic lines were inoculated with the bacterial pathogen *Pst* DC3000 *avrRpt2* at OD 0.0001. The mutant *Arabidopsis cdr1* did not show any HR at 3 days post inoculation or infiltration (DPI) with *Pst* DC3000 *avrRpt2*. On the contrary, wild-type *Arabidopsis* (Col-0) showed a typical HR. Twelve *PtCDR2* transgenic *cdr1* mutant lines and fourteen *PtCDR8* transgenic *cdr1* mutant lines showed the same type of HR as the wild-type *Arabidopsis*. For each *Poncirus* gene, two transgenic *Arabidopsis* lines were selected randomly to record HR on the infiltrated leaves (Figure 2) and to perform subsequent analyses.

### 2.3. PtCDR2 and PtCDR8 Inhibited Pst DC3000 avrRpt2 Growth

**Figure 3:**
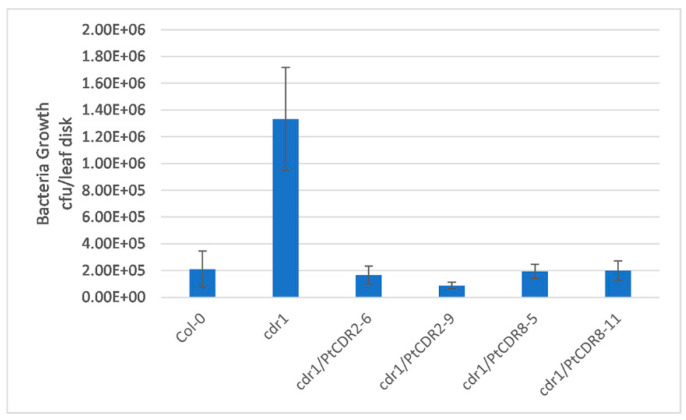
Bacteria growth assay. Twenty-five-day-old *Arabidopsis* plants were inoculated with *Pseudomonas syringae* pv. *tomato* strain DC3000 (*Pst* DC3000) expressing *avrRpt2* by spraying a bacterial cell suspension. Six leaf disks from each plant were collected randomly at 3 DPI, and bacterial cells were extracted and plated out on a solid selective medium containing two antibiotics. Inoculated plants were wild-type *Arabidopsis* (Col-0), *cdr1* mutant (cdr1), and four transgenic *cdr1* lines (cdr1/PtCDR2-6, cdr1/PtCDR2-9, cdr1/PtCDR8-5, and cdr1/PtCDR8-11)

At 3 DPI with *Pst* DC3000 *avrRpt2*, six leaf disks were randomly collected from each *Arabidopsis* plant for bacterial counting. On average, the bacterial count in the wild-type *Arabidopsis* was 6.3-fold less than the bacterial count in the *cdr1* mutant (Figure 3). Bacterial counts in *PtCDR8* transgenic lines 5 and 11 (cdr1/PtCDR8-5 and cdr1/PtCDR8-11) were similar to the bacterial count in the wild-type *Arabidopsis* and reduced by 6.86- and 6.67-fold, respectively, compared to the bacterial count in the *cdr1* mutant. *PtCDR2* transgenic lines 6 and 9 (cdr1/PtCDR2-6 and cdr1/PtCDR2-9) showed stronger bacterial growth inhibition: bacterial counts in these lines were reduced by 8.0- and 15.0-fold, respectively, compared to the bacterial count in the *cdr1* mutant (Figure 3).

### 2.4. PtCDR2 and PtCDR8 Increased the Expression Level of PATHOGENESIS RELATED 1 (PR1) in the cdr1 Mutant

**Figure 4:**
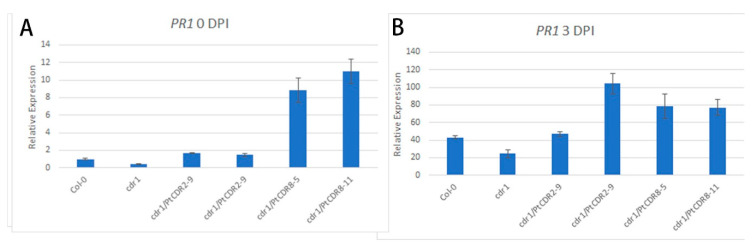
Relative expression of *PR1* in wild-type *Arabidopsis* (Col-0), its *cdr1* mutant (cdr1), and four transgenic lines (cdr1/PtCDR2-6, cdr1/PtCDR2-9, cdr1/PtCDR8-5, and cdr1/PtCDR8-11). Relative expression of *PR1* was determined using the 2^−ΔΔCT^ method. (**A**) Plants were sprayed with *Pseudomonas syringae* pv. *tomato* strain DC3000 (*Pst* DC3000) expressing *avrRpt2* at an OD_600_ 0f 0.001 in 10 mM MgCl_2_ and 0.02% Silwet. Leaf samples were collected at 0 and 3 DPI and total RNA was isolated for quantitative real-time PCR analysis. (**B**) Control plants were sprayed with 10 mM MgCl_2_ and 0.02% Silwet.

Overexpression of *OsCDR1* in *Arabidopsis* led to the upregulation of *PR1* [41], a marker gene in the SA pathway [47]. The *PR1* relative expression level was analyzed among different transgenic lines 3 DPI with *Pst* DC3000 *avrRpt2*. *Arabidopsis* plants in the control were sprayed with a 10 mM MgCl_2_ solution containing 0.02% Silwet L-77. *PR1* expressed at a lower level in the *cdr1* mutant than in the wild-type *Arabidopsis* (Col-0) (0.38-fold) without inoculation of the pathogen (Figure 4A). *PR1* expressed slightly higher in transgenic *PtCDR2* lines 6 and 9 (1.66- and 1.47-fold, respectively) but much higher in *PtCDR8* transgenic lines 5 and 11 (8.86- and 10.99-fold, respectively) (Figure 4A). At 3 DPI, *PR1* expression level was much higher in all genotypes, including the wild type, the *cdr1* mutant, and the four transgenic lines (Figure 4B), suggesting that inoculation with *Pst* DC3000 *avrRpt2* induced *PR1* expression in all genotypes. At this time, *PR1* expression in the *cdr1* mutant was still the lowest and lower than its expression in the wild-type *Arabidopsis*. Different levels of *PR1* upregulation were observed in the transgenic lines: *PtCDR2* line 9 showed the greatest *PR1* upregulation, from 1.47 at 0 DPI to 104.24 at 3 DPI, followed by *PtCDR8* lines 5 and 11 (from 8.66 or 10.99 at 0 DPI to 78.75 or 77.22 at 3 DPI), and *PtCDR2* line 6 (from 1.66 at 0 DPI to 43.12 at 3 DPI) (Figure 4B).

## 3. Discussion

Previous gene expression and genetic mapping studies have indicated that multiple genes could be involved in certain *Poncirus* and *Citrus* cultivars’ resistance/tolerance to HLB [22,28,43,48], but so far very few of these candidate genes have been screened and investigated in depth for their actual roles in HLB resistance/tolerance. The primary impediments leading to the lack of progress in candidate gene screening and gene function confirmation are the existence of substantial difficulties in the production of transgenic citrus plants for these candidate genes, inoculation of the transgenic plants with *C*Las, and collection of reliable *C*Las bacterial titers and HLB symptom severity scores from the inoculated transgenic plants. Alternate systems are much needed to circumvent some of these challenges and to screen multiple candidate genes to identify the best ones for use in engineering citrus for HLB resistance. Facing similar difficulties with studying *C*Las in citrus, plant pathologists have made use of the model plant *Nicotiana benthamiana* to express candidate *C*Las effector genes and understand their roles in *C*Las pathogenesis [6]. Recently, *Nicotiana benthamiana* was also experimentally infected with *C*Las via dodder transmission and used to identify a critical *C*Las effector LasΔ5315 and determine its role in the development of prominent HLB symptoms, including starch accumulation and leaf chlorosis [49]. Previously, the garden flower periwinkle (*Catharanthus roseus*) was infected with *C*Las, and the infected periwinkle was used to screen antibiotics and various other chemical compounds for controlling or suppressing *C*Las in planta [50]. We considered using *Nicotiana benthamiana* and periwinkle for testing the potential role of *PtCDR2* and *PtCDR8* in plant disease resistance. *Nicotiana benthamiana* can be readily transformed by co-cultivation of leaf discs with *Agrobacterium*, and transgenic lines can be inoculated with *C*Las via dodder transmission [49]. However, information was not available regarding *Nicotiana benthamiana* resident *CDR1* or *CDR1*-like genes, and desired *cdr1* mutants were not available. Periwinkle is extremely difficult to transform with *Agrobacterium*, and no information was available about its resident *CDR1* or *CDR1*-like genes either. This situation prompted us to explore the use of *Arabidopsis* mutants as an alternate system to test *PtCDR2* and *PtCDR8* and to determine if they would function in *Arabidopsis* and play any role in disease resistance. 

Our results described above clearly showed that both *PtCDR2* and *PtCDR8* restored the function of *Arabidopsis CDR1* and conferred the *cdr1* mutant HR and resistance to the bacterial pathogen *Pst* DC3000 expressing *avrRpt2*. Our study indicated that *Arabidopsis* mutants could serve as a useful proxy for screening candidate genes and confirming their role in disease resistance. Using proper *Arabidopsis* mutants may offer a number of advantages for screening candidate disease resistance and defense genes, as shown in this study. Foreign genes can be readily introduced into *Arabidopsis* mutants by simple floral dip procedures. Sufficient numbers of homozygous transgenic lines could be produced within several months by selfing T0 transgenic plants. More importantly, a huge collection of *Arabidopsis* mutants, including numerous mutants with their resident disease resistance and defense genes disrupted, are readily available for use in both forward and reverse genetic studies [51]. *Arabidopsis* mutants have especially helped formulate the concept of the plant immune system and reveal various plant disease immunity signaling pathways and key genetic factors in these pathways [52]. This study may represent the first effort in using *Arabidopsis* mutants to screen *Poncirus* genes for their potential roles in disease resistance. Our study demonstrated the value of *Arabidopsis* mutants in such an effort. We believe that these mutants may play even more important roles in future citrus genetic studies toward identifying candidate genes for engineering citrus for HLB resistance when *C*Las cultures become available. 

Our results showed that *PtCDR2* and *PtCDR8* induced significant upregulation of *PR1* after pathogen inoculation. The induced *PR1* upregulation may indicate the potential value of expressing *PtCDR2* and *PtCDR8* for enhancing HLB resistance in citrus. *PR1* is an inducible marker gene for the SA-mediated plant defense system and plays key roles in plant systemic acquired resistance (SAR) to diseases. In a recent study in citrus, the level of *PR1* expression in transgenic sweet orange cultivars ‘Hamlin’ and ‘Valencia’ was directly co-related to the *Arabidopsis NPR1*-mediated resistance to HLB, whereas the expression of the transgene *AtNPR1* itself in citrus was not directly co-related to HLB resistance [33]. A similar phenomenon was observed in rice and *Arabidopsis* transformed with rice *CDR1* (*OsCDR1*): *OsCDR1*-enhanced disease resistance in transgenic rice and *Arabidopsis* was also correlated with the induction of *PR1* [41]. Based on the observed relationship between *PR1* expression and enhanced disease resistance, especially HLB resistance in *AtNPR1*-transgenic sweet orange, it seems reasonable to speculate that overexpression of *PtCDR2* and *PtCDR8* may result in similar upregulation of *PR1* in transgenic sweet orange and, thus, similarly enhanced HLB resistance. On the other hand, PtCDR2 and PtCDR8 are predicted to be extracellular proteins, like OsCDR1 [41]. This may raise a question: How could PtCDR2 and PtCDR8 as predicted extracellular proteins impart citrus resistance/tolerance to *C*Las, a bacterial pathogen residing inside citrus phloem elements? Considering these aspects, we speculate that the functioning of PtCDR2 and PtCDR8 may involve some intriguing pathways or pathway components to activate the plant defense system. It has been hypothesized that OsCDR1 activation can lead to the generation of an endogenous extracellular peptide elicitor, and the released elicitor can rapidly activate basal local and systemic defense responses [40,41]. To answer these questions in citrus, we initiated an effort to introduce these genes into sweet orange and produce transgenic lines. The transgenic sweet orange lines will be inoculated with *C*Las to determine their resistance to HLB. 

Overexpression of *OsCDR1* in rice resulted in enhanced resistance to *Xanthomonas oryzae* and *Magnaporthe oryzae*, the bacterial pathogen of rice blight and the fungal pathogen of rice blast [41]. Overexpression of the same gene in *Arabidopsis* led to increased resistance against infection by bacterial pathogen *Pst* DC3000 and fungal pathogen *Hyaloperonospora arabidopsidis* but not against the necrotrophic pathogen *Alternaria brassicicola* [41]. These previous studies seem to indicate potential of using *PtCDR2* and/or *PtCDR8* for engineering resistance to multiple pathogens. In citrus, the bacterial pathogen *Xanthomonas citri* ssp. *citri* (*Xcc*) causes citrus canker, a disease that is important and also difficult to control [53]. Natural genetic resistance to *Xcc* is rare in citrus. Transgenic resistance to *Xcc* has been pursued for many years [54,55]. It will be very interesting to find out if *PtCDR2* and *PtCDR8* transgenic sweet orange will have increased resistance to *Xcc*.

In summary, we have shown that *Arabidopsis* mutants (in this case, the *cdr1* mutant) can serve as a useful alternate system for screening *Poncirus* (and *Citrus*) genes for their roles in plant defense and that *PtCDR2* and *PtCDR8* both are functional genes and play a key role in plant defense responses. These genes may serve as strong candidate genes for engineering citrus for disease resistance, including resistance to HLB, the deadliest bacterial disease of citrus worldwide.

## 4. Materials and Methods

### 4.1. Plant Materials

The *Poncirus trifoliata* plants (accession DPI 50-7) were kept in a greenhouse with natural light. *Arabidopsis* plants were grown in a growth room with a 16 h light/8 h dark cycle at 21 ℃. The *cdr1* mutant (stock number SALK_050514) and wild-type *Arabidopsis* Col-0 (stock number CS70000) were purchased from TAIR. 

### 4.2. Sequence Analysis

Protein sequence alignment and phylogenetic analysis were performed with the software Mega X [56]. The neighbor-joining method was used to generate a phylogenetic tree. The protein sequences of AtCDR1, CsCDR1, and CcCDR1 were retrieved from NCBI [57,58]; other CDRs from *Citrus maxima* (Cg6g008160.1), *Citrus medica* (Cm260950.1), *Citrus reticulata* (MSYJ114420.1), and *Atalantia buxifolia* (sb29852.1) were retrieved from the *Citrus sinensis* annotation project [59]. 

### 4.3. Gene Cloning, Construction of Expression Vectors, and Transformation of Arabidopsis cdr1 Mutant

Mature leaves were collected from *P. trifoliata* plants grown in containers in the greenhouse. Total RNA was extracted from the leaves using the RNeasy plant mini kit (Qiagen, Cat. 74904). RNAs were reverse transcribed to cDNA using the SuperScript™ III First-Strand Synthesis System (Thermofisher Scientific, Cat. 18080051) and oligo(dT). *PtCDR2* and *PtCDR8* were amplified with Phusion^®^ High-Fidelity DNA Polymerase (NEB, Cat. M0530S) and primers PtCDR F (CCCGGGATGGCAACCTTCTTGAGTTGTGC) and PtCDR2 R (GAGCTCCAAAATTTAATTACAGCTTGGTGC) or PtCDR F and PtCDR8 R (GAGCTCGCTTCCCAATTAATTATTGCTTGGTGC). The amplified DNA fragments were purified after treatment with *Taq* polymerase and cloned into the pGEM-T Easy vector (Promega, Cat. A1360). The plasmid containing *PtCDR2* or *PtCDR8* was digested with *Xma*I and *Sac*I, and then the *PtCDR2* and *PtCDR8* fragments were cloned into the expression vector pCAMBIA1300-221 [60]. *PtCDR2* and *PtCDR8* were driven by the CaMV 35S promoter and terminated by the Nos terminator. These expression vectors were then introduced into the *Agrobacterium tumefaciens* strain EHA105 by electroporation. The *Arabidopsis cdr1* mutant was transformed by floral dip [61]. T0 transgenic seeds were selected on MS plates containing kanamycin (50 mg/L) and hygromycin (20 mg/L). The positive seedlings were transplanted into soil for disease assay. 

### 4.4. Disease Assay

The model pathogen *Pseudomonas syringae* pv. *tomato* strain DC3000 (*Pst* DC3000) expressing *avrRpt2* was used in this research. Bacteria were cultured at 28℃ on a shaker in the King’s B medium containing two antibiotics, rifampin (20 mg/L) and kanamycin (50 mg/L). When the optical density (OD_600_) of the bacterial culture reached 0.6, bacterial cells were harvested by centrifugation and resuspended in a 10 mM MgCl_2_ solution. The fifth to seventh rosette leaves of 25-day-old *Arabidopsis* plants were infiltrated with *Pst* DC3000 *avrRpt2* at an OD_600_ of 0.0001 (5 × 10^4^ CFU/mL) using a 1 mL needleless syringe. The inoculated leaves were photographed at 3 DPI. For the bacterial growth assay, the 25-day-old *Arabidopsis* plants were sprayed with a *Pst* DC3000 *avrRpt2* bacterial suspension at an OD_600_ of 0.001 (5 × 10^5^ CFU/mL) and containing 0.02% Silwet L-77. Six leaf disks were randomly collected from six plants of each genotype with a core borer (6 mm in diameter). The leaf disks were ground, and the ground tissues were diluted serially by an increment of 10 and plated on solid King’s B medium containing rifampin (20 mg/L) and kanamycin (50mg/L). The plates were incubated at 28 ℃ in the dark for two days before bacterial colonies were counted. 

### 4.5. Gene Expression Analysis

The rosette leaves of *Arabidopsis* were collected three days after the plants were spray-inoculated with a *Pst* DC3000 bacterial suspension. RNAs were extracted from the collected *Arabidopsis* leaf samples using an RNeasy Plant Mini kit (Qiagen, Cat. 74904), following the manual exactly. A High-Capacity RNA-to-cDNA™ Kit (ThermoFisher Scientific, Cat. 4387406) was used for reverse transcription according to the manufacturer’s instruction. Quantitative real-time PCR was performed using PowerUp™ SYBR™ Green Master Mix (ThermoFisher Scientific, Cat. A25742) and the QuantStudio 5 real-time PCR system (ThermoFisher Scientific) according to the manufacturer’s specifications. The *Actin 2* gene was used as the internal reference, and it was amplified with a forward primer 5′- GATCTCCAAGGCCGAGTATG-3′ and a reverse primer 5′- CCCCAGCTTTTTAAGCCTTTG-3′. Defense marker gene *PR1* was analyzed with primers PR1F 5′-CTCATACACTCTGGTGGG-3′ and PR1R 5′- ATTGCACGTGTTCGCAGC-3′. The relative expression of *PR1* was calculated using the 2^−ΔΔCT^ method [62].
